# Seasonal UV exposure and vitamin D: association with the dynamics of COVID‐19 transmission in Europe

**DOI:** 10.1002/2211-5463.13309

**Published:** 2021-12-04

**Authors:** Sunanda Biswas Mukherjee, Alessandro Gorohovski, Eugene Merzon, Eliad Levy, Sumit Mukherjee, Milana Frenkel‐Morgenstern

**Affiliations:** ^1^ Cancer Genomics and BioComputing of Complex Diseases Lab Azrieli Faculty of Medicine Bar‐Ilan University Safed Israel; ^2^ Leumit Health Services Tel Aviv Israel; ^3^ Department of Family Medicine Sackler School of Medicine Tel Aviv University Israel

**Keywords:** COVID‐19, seasonality, ultraviolet index, cloud‐free vitamin D UV doses, plasma 25(OH) vitamin D

## Abstract

Several recent studies have demonstrated that low plasma 25(OH) vitamin D levels are associated with the risk of COVID‐19 infection. The primary source of vitamin D production in humans is environmental UV radiation. In many viral respiratory diseases, peak infection rates are observed during winter due to reduced UV exposure and low temperatures. In Europe, the second wave of COVID‐19 began early in the winter of 2020. Investigating the impact of seasonal temperature and UV exposure on COVID‐19 transmission could thus aid in prevention and intervention. As such, we first performed a comprehensive meta‐analysis of all related published literature based on the association between vitamin D and COVID‐19, which supported the hypothesis that the low vitamin D level is a critical risk factor for COVID‐19 infection. Next, to understand the potential impact of seasonal UV and temperature levels on COVID‐19 cases, we analyzed meteorological data and daily COVID‐19 cases per million in the populations of 26 European countries. We observed that low temperature, UV index, and cloud‐free vitamin D UV dose (UVDVF) levels are negatively correlated with COVID‐19 prevalence in Europe. Furthermore, a distributed lag nonlinear model was used to assess the nonlinear delayed effects of individual seasonal factors on COVID‐19 cases. Such analysis highlighted the significantly delayed impact of UVDVF on the cumulative relative risk of COVID‐19 infection. The findings of this study suggest that low UV exposure can affect the required production of vitamin D in the body, which substantially influences the dynamics of COVID‐19 transmission and severity.

## Graphical abstract

We observed a strong correlation of UV exposure with COVID‐19 incidences for 26 European countries. Exposure–lag–response association analysis revealed that UV exposure had a significant delayed effect on COVID‐19 incidences. Meta‐analysis of vitamin D levels in COVID‐19 suggests that low vitamin D level is a critical risk factor for COVID‐19. In conclusion, low UV exposure could affect the required vitamin D production in the body, which substantially impacts COVID‐19 transmission.

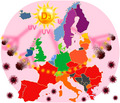

**Abbreviations**
DLNM: distributed lag nonlinear model
RR: relative risk
UV index: ultraviolet index
UVDVF: cloud‐free vitamin D UV doses

Most viral respiratory infections follow seasonal patterns, with high numbers of cases appearing during winter [1, 2, 3]. For instance, influenza virus and respiratory syncytial virus (RSV) infections follow this trend, showing annual seasonal peaks during winter, namely from December to March in the Northern Hemisphere, and from June to August in the Southern Hemisphere [4]. Likewise, epidemiological studies of common cold‐causing human coronavirus (HCoV) infections suggest that these also follow a seasonal pattern, whereby HCoV infections are mainly detected in winter and spring in several countries [5, 6, 7, 8, 9, 10, 11]. The ongoing COVID‐19 pandemic is caused by SARS‐CoV‐2, which is a highly contagious virus that is associated with severe respiratory illness [12, 13, 14, 15]. It is still under debate whether seasonality can explain the transmission dynamics of COVID‐19 [16], although several studies have proposed that COVID‐19 might indeed follow a seasonal pattern [17, 18, 19]. Early studies conducted at the onset of the COVID‐19 outbreak predicted that patterns of disease transmission would be consistent with the behavior of a seasonal respiratory virus [20]. Recent studies based on 40 days' worth of data demonstrated that sunlight exposure is associated with COVID‐19 recovery rates in Jakarta, Indonesia [21]. Based on data collected in the first 4 months of the pandemic, another recent study demonstrated that COVID‐19 transmission rates are associated with UV light, temperature, and relative humidity [22]. Further experimental studies also suggested that SARS‐CoV‐2 is sensitive to these same parameters [23]. However, it is difficult to draw inferences based on such studies, as these analyses were performed in the early stages of viral spread and are based on only 3–4 months' worth of data. As the Northern Hemisphere moved into winter (2020/21), several European countries experienced a second wave of COVID‐19 infection [24, 25]. Therefore, it is necessary to explore the impact of seasonal factors, particularly UV and temperature levels, on the surge of COVID‐19 cases in Europe.

In the present study, we analyzed the association of daily COVID‐19 transmission with average daily temperature, ultraviolet index (UV index), and cloud‐free vitamin D UV doses (UVDVF) in 26 European countries. We observed that temperature, UV, and UVDVF are negatively correlated with the number of daily COVID‐19 cases in these countries. Furthermore, exposure–lag–response association analysis using a distributed lag nonlinear model (DLNM) revealed that the increased UVDVF experienced on different lag days is significantly associated with a decreased relative risk (RR) of COVID‐19. In humans, UVDVF absorbed by the skin serves as the main inducer of vitamin D production [26]. Vitamin D synthesis occurs when the skin is exposed to sunlight, which varies seasonally, such that seasonality modulates the intensity of UV radiation. Previous studies demonstrated that UV radiation varies considerably with the seasons across the globe, with both UV radiation and temperature being moderators of respiratory viral epidemiology [2, 27]. It was also reported that the seasonality of respiratory viruses alters susceptibility by changing serological vitamin D levels [28]. Vitamin D plays an immunomodulatory role in the body, and it was reported that low vitamin D levels are associated with increased susceptibility to COVID‐19 [29, 30, 31, 32, 33, 34]. Therefore, to evaluate the association between vitamin D deficiency and COVID‐19 infection, we carried out a meta‐analysis based on available literature and found that vitamin D deficiency is associated with a higher risk of contracting COVID‐19. Hence, our results suggest that the extent of UV radiation and the ensuing levels of vitamin D in the body are important factors for COVID‐19 transmission.

## Methods

### Text‐mining and meta‐analysis

Available text‐mining tools, such as those found at LitCovid [35], PubTator [36], and the iSearch platform (https://icite.od.nih.gov/covid19/search/), were used to extract manuscripts related to vitamin D and COVID‐19. The word forms ‘Vitamin D’ and ‘Vitamin D deficiency’, as well as any alternative words found in the MeSH, were used [37]. The logical operators used were ‘AND’ and ‘OR’ as a combination of handles to search for different publications. After extensive screening, twelve articles were selected for the meta‐analysis. The meta‐analysis was performed using CMA software and MedCalc [38]. Minimum and maximum serum 25(OH) vitamin D levels were listed according to the values reported in each article. To assess the outcomes, we used the risk ratios and their associated 95% confidence intervals, with a *P* value < 0.05 considered as being statistically significant. We assessed heterogeneity using the *I* ^2^ test. Publication bias was evaluated using funnel plots.

### Collection of daily COVID‐19 cases and meteorological data for European countries

For the period from March 1, 2020, to December 20, 2020, we downloaded the ‘number of daily new COVID‐19 cases (per million of the population)’ and ‘7‐day average of daily new cases (per million of the population)’ from different European countries from the ‘Our World in Data’ web portal (https://ourworldindata.org/coronavirus). As the number of daily new COVID‐19 cases depends on the number of COVID‐19 tests and updates in the database, there is the chance that this information may sometimes not be accurate for certain days. To overcome this issue, we also used data under the ‘7‐day average of daily new cases’ heading, which can reflect the overall case statistics for a given day. The daily UV index and UVDVF were collected from UV station data based on listings at the operational TEMIS satellite ozone data web portal (http://www.temis.nl/uvradiation/UVarchive/stations_uv.html), while daily temperatures were obtained from the daily summaries found at the National Oceanic and Atmospheric Administration (NOAA) web portal (https://www.ncdc.noaa.gov/cdo‐web/datasets). Based on the location of the different UV‐ and temperature‐monitoring stations available at the TEMIS and NOAA web portals, we identified 87 UV stations in 26 European countries that also collected temperature data. The average UV index, UVDVF, and temperature from the different stations in a country were used for further analysis. Finally, we generated a dataset listing meteorological factors and daily COVID‐19 case numbers for 26 European countries (Table S1).

### Correlation analysis

We performed Pearson's correlation analysis to analyze the correlation between meteorological parameters (UV index, UVDVF, and average temperature) and COVID‐19 case numbers so as to understand the linear relationship between these factors. The Pearson correlation coefficient (*r*) between each meteorological factor and COVID‐19 case numbers was calculated using the ‘SPSS Package’ [39] with a threshold of significance (*P* = 0.05).

### Distributed lag nonlinear model for exposure–lag–response association analysis

To quantitatively analyze the effects of different meteorological predictors (temperature, UV, and UVDVF) on COVID‐19 cases, a generalized linear model (GLM) and a DLNM were used [40, 41]. To assess the influence of the selected predictors on COVID‐19 cases, we performed a simulation study based on scenarios of (dose of meteorological predictor)–(lag)–(response) surfaces. The RR of COVID‐19 was used to represent the impact of a set of meteorological predictors.

In our study, a GLM assumed that linear and nonlinear variables could be combined in the form of a sum, in which the effects of the nonlinear variables can be represented by nonlinear function terms. Smooth functions (B‐spline, natural cubic spline) were used for fitting the independent variables. The model can be written as:

(1) $$\begin{array}{ll} \log\left[E\left(y_{t}\right)\right]= & \;\alpha \;+\;s\left({\text{Temp}}_{t},\ldots ,{\text{Temp}}_{t‐21}\right)\;\\ & +\;s\left({\text{UV}}_{t},\ldots ,{\text{UV}}_{t‐21}\right)\;\\ & +\;s\left({\text{UVDVF}}_{t},\ldots ,{\text{UVDVF}}_{t‐21}\right)\;+\;\text{dow;} \end{array}$$

$$\text{dow}\;=\;g\left(t,\gamma \right)\;+\;\sum_{j=1}^{6}\delta_{j}\omega_{j,t};\;\forall t\;=\;1,\ldots ,295.$$

where log is used as a link function (quasi‐Poisson GLM); *E(y_t_)* is the number of events with COVID‐19 cases; α represents residuals of the GLM; *s*() denotes quadratic B‐spline functions defined by two equally spaced internal knots for nonlinear variables; *Temp, UV, and UVDVF* predictors represent temperature, UV, and UVDVF, respectively. These predictors were represented by the daily average temperature, UV, and UVDVF series in different countries within the period spanning 2020‐03‐01 to 2020‐12‐20 (295 days); *dow* (day of the week) is a natural cubic spline of vector from Monday to Saturday (Sunday is the reference level) defined by 7 df/year, accounting for seasonal and long‐term trends, while *w_j_* is an indicator of the day of the week.

The DLNM has long been used to describe the lagged effect of meteorological predictors on health outcomes [40]. It uses a cross‐basis function to describe a two‐dimensional relationship along the dimensions of meteorological factors and lag. The choice of cross‐basis functions for the meteorological element and lag is independent, such that spline or linear functions can be used, while polynomial functions can be used for the lag. The resulting estimates can be plotted as a three‐dimensional graph to show the RRs for both meteorological elements and lags. Cross‐basis functions for the meteorological factors (daily average temperature and UVDVF) were established in this study. These effects were estimated using nonlinear smoothing functions for both dimensions, in which a natural cubic spline was used for each meteorological element and a polynomial spline for the lag effect.

To avoid singularity of the model, it is necessary to avoid too many nonlinear terms. In our study, therefore, we examined only two meteorological predictors. Moreover, since UVDVF is computed from UV, we excluded UV from the model. Thus, the final model, with a relationship between counts of all‐cause COVID‐19 cases on day *t* and temperature, accounting for up to 21 days of lag, and a quasi‐Poisson GLM, can be written as follows:

$$\begin{array}{ll} \log\left[E\left(y_{t}\right)\right]= & \alpha \;+\;cb\left({\text{Temp}}_{t},\ldots ,{\text{Temp}}_{t‐21};\eta \right)\;\\ & +\;cb\left({\text{UVD}}_{t},\ldots ,{\text{UVD}}_{t‐21};\eta \right)\;\\ & +\;\text{dow};\forall t\;=\;1,\ldots ,295. \end{array}$$

where *cb()* denotes the cross‐basis functions for nonlinear variables, with *η* corresponding to coefficients of the cross‐basis and *γ* to coefficients of additional covariate terms in the model, respectively. Other terms are defined as in Eqn (1).

Using this GLM, we also calculated the RR, which is defined as the ratio of the probability of COVID‐19 developing in a group exposed to a specified environment to the probability of the disease developing in a nonexposed control group. The RR (representing the risk of casualties caused by a unit change in environmental conditions) was used to quantify the impact of meteorological predictors on COVID‐19 patients. The range of RR is (0, ∞); RR = 1 means no connection between exposure to an environmental condition and disease; RR < 1 means that exposure resulted in a reduction of the incidence of the disease (i.e., exposure is a protective factor); and RR > 1 means the opposite. The corresponding 95% confidence interval was calculated using the equation:

$$95\%\text{CI}\;=\;\exp\left[\left(\beta \;\pm \;1.96\;\text{SE}\right)\;\times \;\text{dt}\right].$$

The analyses were performed using the r version 3.6.3 (2020‐02‐29) software (R Foundation for Statistical Computing, Vienna, Austria. https://www.R‐project.org/) environment. The ‘mgcv’ program package (version 1.8‐31, https://cran.r‐project.org/web/packages/dlnm/index.html) was used for the GLM, and the ‘dlnm’ program package (version 2.4.5, https://cran.r‐project.org/web/packages/dlnm/index.html) was used for the DLNM [42].

## Results

### Low vitamin D levels and susceptibility to COVID‐19 infection: a meta‐analysis of observational studies

**Fig. 1:**
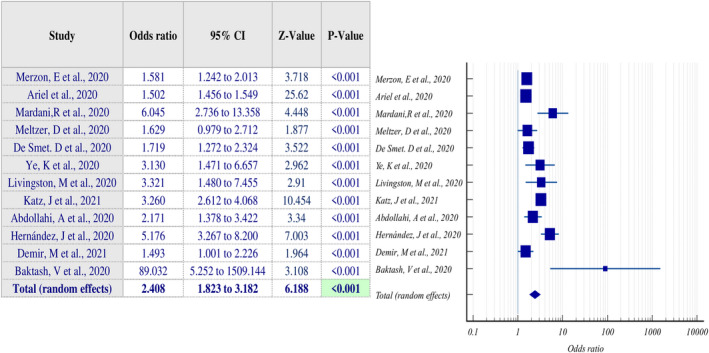
Meta‐analysis of observational studies evaluating the association between vitamin D deficiency and susceptibility to COVID‐19.

**Fig. 2:**
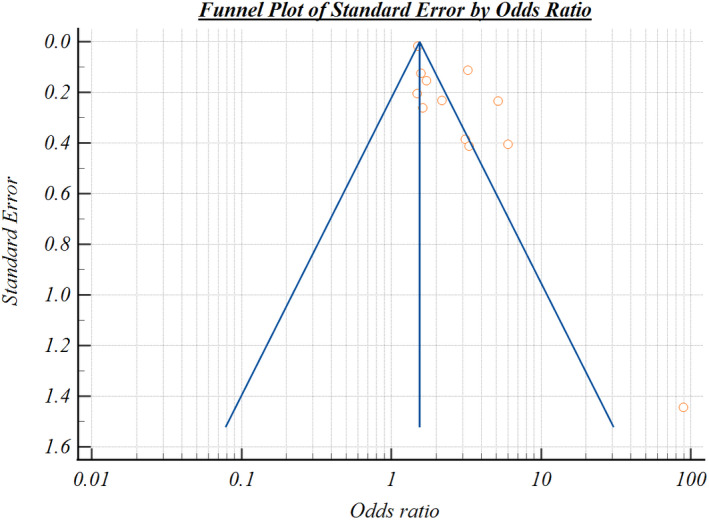
Funnel plot of the association between vitamin D deficiency and COVID‐19 case studies.

After extensive literature searches using a text‐mining approach [43], we found twelve articles suitable for our analysis. These papers reported comparisons of plasma 25(OH) vitamin D levels in COVID‐19 patients with controls. In these twelve articles [31, 33, 44, 45, 46, 47, 48, 49, 50, 51, 52, 53], plasma 25(OH) vitamin D levels were assessed in a total of 1,577,260 subjects, out of which 55,198 subjects were COVID‐19‐positive patients, and 1,522,062 subjects were COVID‐19‐negative individuals. We employed a random‐effects model of meta‐analysis as we assumed that the observed estimates of plasma 25(OH) vitamin D levels could vary across studies due to differences in the subjects studied and their locations. Our meta‐analysis results showed that a low mean plasma 25(OH) vitamin D level is significantly associated with higher susceptibility to COVID‐19 infection (odds ratio (OR) 2.408, 95% confidence interval (CI) 1.823 to 3.182, *P* < 0.001; Fig. 1). A test of heterogeneity showed that there was moderate heterogeneity among the studies (*I* ^2^ = 89.24%, *P* < 0.001), meaning that a significant combination of the variability of the data existed in the analysis. Moreover, the funnel plot generated from the data shows the significant distribution, suggesting the studies included in the study were significant (Fig. 2). Thus, our meta‐analysis results suggest that a low plasma 25(OH) vitamin D level is a critical risk factor for being susceptible to COVID‐19.

### Daily COVID‐19 cases are negatively associated with temperature, UV index, and UVDVF in different European countries

**Fig. 3:**
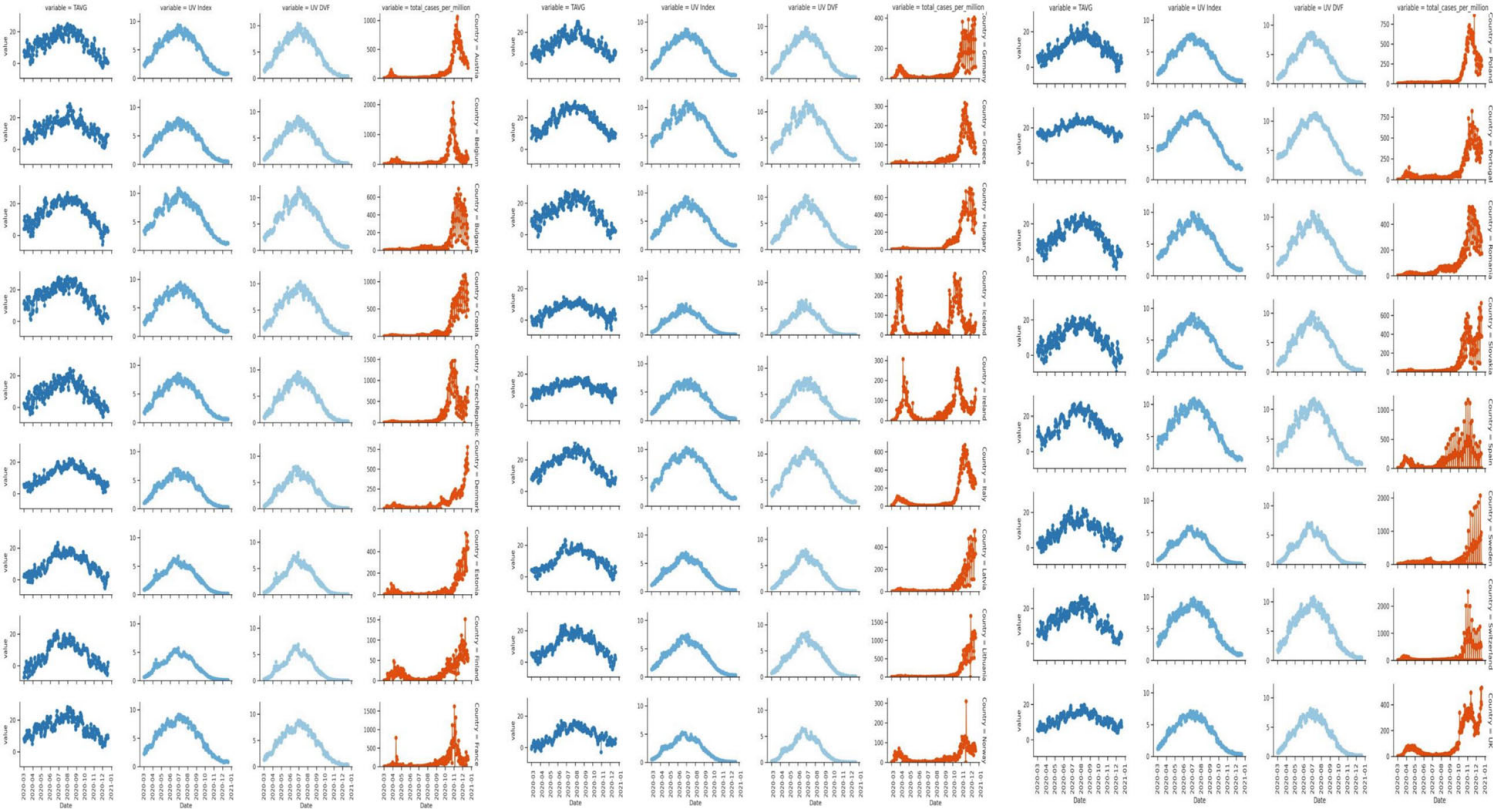
Temperature, UV, UVDVF, and daily COVID‐19 case numbers in different European countries from March to December 2020.

The appearance of new COVID‐19 cases has continued to increase in European countries. We collected meteorological data and daily COVID‐19 case numbers from 26 European countries over 10 months. Interestingly, we observed that the number of cases was low during the peak summer season (June–August 2020). At the start of autumn, several European countries began experiencing a second wave of COVID‐19 transmission, with France, the UK, and Spain reporting record numbers of new cases in these months. Moreover, the number of new cases in the UK and several other European countries, including Germany and Italy, increased at a significantly higher rate than in earlier months. In the last 2 weeks of October 2020, the number of COVID‐19 cases jumped from 7 million to 9 million. The monthly average number of coronavirus cases and the corresponding monthly temperature, UV index, and UVDVF are presented in Fig. 3.

**Fig. 4:**
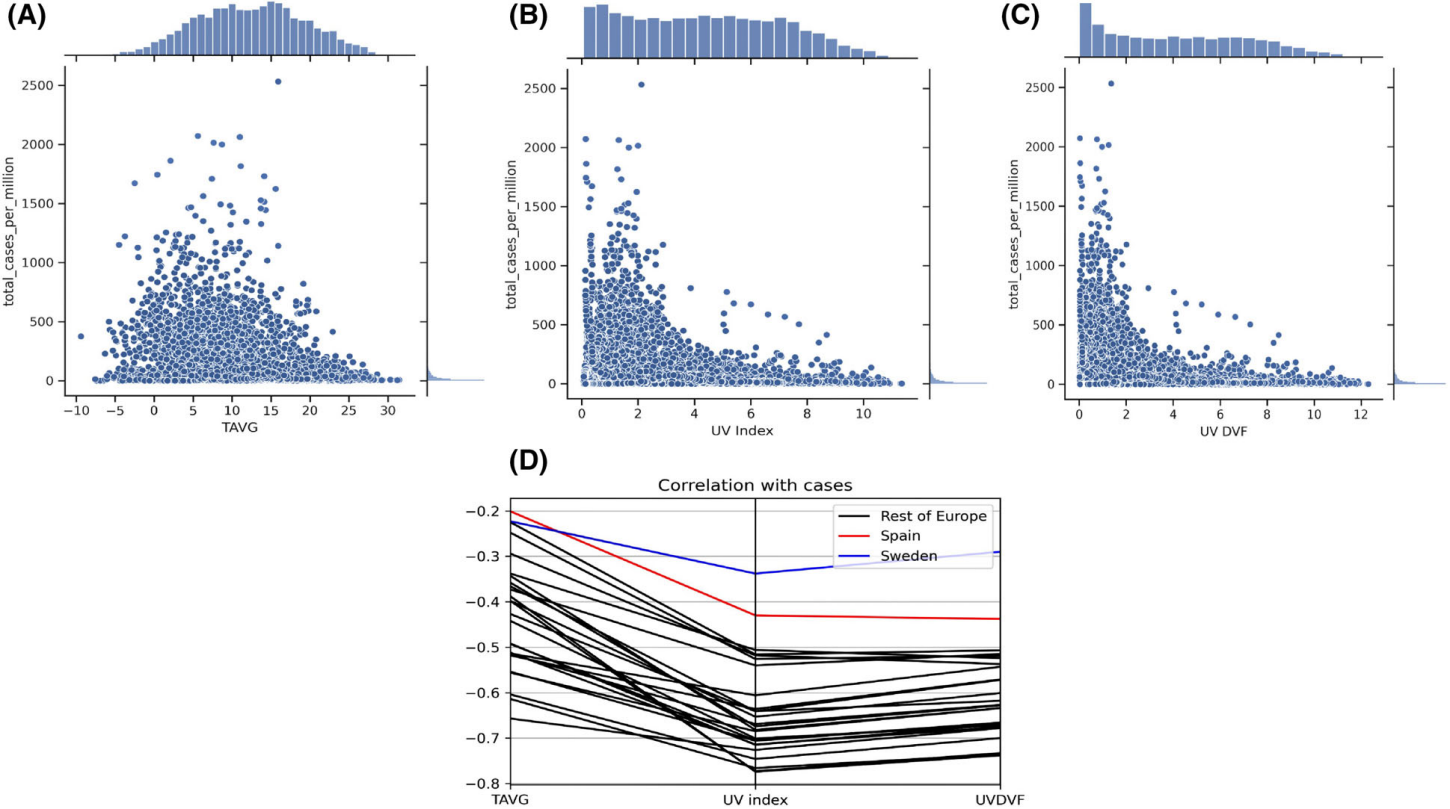
Trend of association of the number of COVID‐19 cases with (A) daily average temperature, (B) UV index, and (C) UVDVF in Europe. (D) Parallel coordinates plot of the Pearson correlation coefficients for COVID‐19 cases and meteorological factors from Table 1.

To understand whether there is any relation between COVID‐19 case numbers and seasonality in Europe, we performed a Pearson correlation analysis of the ‘number of daily new COVID‐19 cases’ and ‘7‐day average of daily new cases’ with temperature, UV index, and UVDVF. The results highlighted that both ‘number of daily new COVID‐19 cases’ and ‘7‐day average of daily new cases’ showed a significant negative association with temperature, UV, and UVDVF in all of the European countries addressed (Table 1). However, the ‘7‐day average of daily new cases’ showed a better correlation coefficient than the ‘number of daily new COVID‐19 cases’, which is sometimes not recorded or properly updated. Figure 4A–C shows the trend of association of temperature, UV index, and UVDVF with COVID‐19 case numbers in Europe. One can observe that the number of COVID‐19 transmissions decreased significantly as the UV index, UVDVF, and temperature increased. The parallel coordinate plots of Pearson's correlation coefficients (Fig. 4D) highlight how UV index and UVDVF have the strongest negative correlations with daily COVID‐19 case numbers in the European countries. Thus, our results indicate that low UV is the important factor for determining the prevalence of COVID‐19 instances in Europe.

**Table 1:** Pearson's correlation analysis of the ‘number of daily new COVID‐19 cases’ and ‘7‐day average of daily new cases’ with temperature, UV index, and UVDVF.

Country	Pearson's correlation coefficients					
	Average temperature		UV index		UVDVF	
	Daily new cases	7‐day average of daily new cases	Daily new cases	7‐day average of daily new cases	Daily new cases	7‐day average of daily new cases
Austria	−0.554^a^	−0.588^a^	−0.706^a^	−0.725^a^	−0.667^a^	−0.682^a^
Belgium	−0.225^a^	−0.241^a^	−0.516^a^	−0.556^a^	−0.507^a^	−0.541^a^
Bulgaria	−0.515^a^	−0.577^a^	−0.669^a^	−0.733^a^	−0.627^a^	−0.685^a^
Croatia	−0.657^a^	−0.687^a^	−0.726^a^	−0.750^a^	−0.678^a^	−0.699^a^
Czech Republic	−0.366^a^	−0.385^a^	−0.641^a^	−0.685^a^	−0.618^a^	−0.657^a^
Denmark	−0.398^a^	−0.399^a^	−0.653^a^	−0.666^a^	−0.601^a^	−0.613^a^
Estonia	−0.514^a^	−0.553^a^	−0.606^a^	−0.629^a^	−0.543^a^	−0.564^a^
Finland	−0.556^a^	−0.593^a^	−0.685^a^	−0.716^a^	−0.634^a^	−0.660^a^
France	−0.248^a^	−0.352^a^	−0.526^a^	−0.665^a^	−0.519^a^	−0.651^a^
Germany	−0.492^a^	−0.543^a^	−0.715^a^	−0.778^a^	−0.670^a^	−0.728^a^
Greece	−0.442^a^	−0.477^a^	−0.701^a^	−0.733^a^	−0.672^a^	−0.699^a^
Hungary	−0.604^a^	−0.639^a^	−0.746^a^	−0.762^a^	−0.700^a^	−0.713^a^
Iceland	−0.338^a^	−0.347^a^	−0.506^a^	−0.536^a^	−0.524^a^	−0.555^a^
Ireland	−0.294^a^	−0.319^a^	−0.518^a^	−0.538^a^	−0.537^a^	−0.552^a^
Italy	−0.614^a^	−0.634^a^	−0.766^a^	−0.772^a^	−0.736^a^	−0.738^a^
Latvia	−0.427^a^	−0.482^a^	−0.641^a^	−0.680^a^	−0.572^a^	−0.605^a^
Lithuania	−0.518^a^	−0.554^a^	−0.636^a^	−0.668^a^	−0.571^a^	−0.598^a^
Norway	−0.358^a^	−0.425^a^	−0.674^a^	−0.748^a^	−0.628^a^	−0.696^a^
Poland	−0.343^a^	−0.367^a^	−0.682^a^	−0.695^a^	−0.634^a^	−0.643^a^
Portugal	−0.388^a^	−0.443^a^	−0.774^a^	−0.798^a^	−0.738^a^	−0.758^a^
Romania	−0.512^a^	−0.572^a^	−0.714^a^	−0.753^a^	−0.676^a^	−0.710^a^
Slovakia	−0.493^a^	−0.539^a^	−0.705^a^	−0.766^a^	−0.666^a^	−0.723^a^
Spain	−0.201^a^	−0.339^a^	−0.430^a^	−0.712^a^	−0.438^a^	−0.720^a^
Sweden	−0.223^a^	−0.376^a^	−0.338^a^	−0.549^a^	−0.290^a^	−0.467^a^
Switzerland	−0.373^a^	−0.545^a^	−0.540^a^	−0.746^a^	−0.515^a^	−0.709^a^
UK	−0.398^a^	−0.406^a^	−0.773^a^	−0.780^a^	−0.733^a^	−0.737^a^

^a^ Represents correlation significant at the 0.01 level.

### Exposure–lag–response association of individual seasonal factors with COVID‐19 cases

**Fig. 5:**
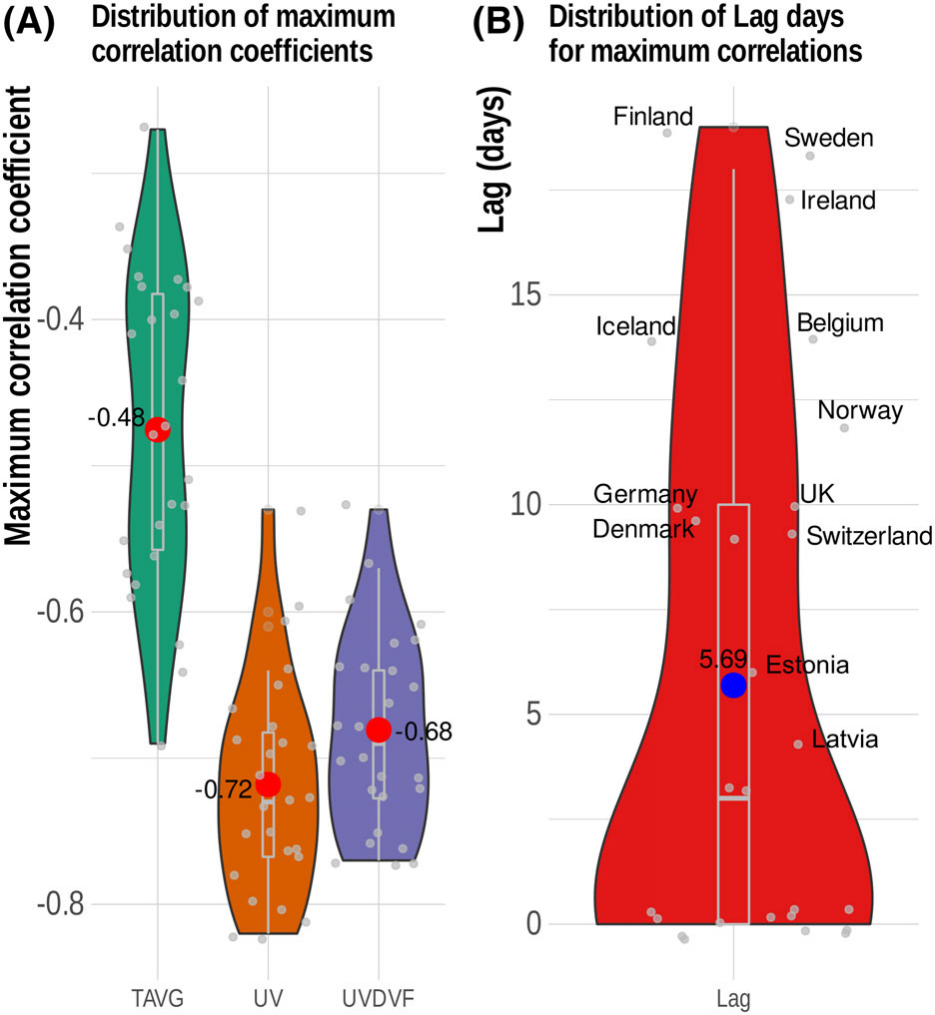
(A) Distribution of maximum Pearson's correlation coefficients for temperature, UV, and UVDVF versus COVID‐19 cases for all countries obtained from different lags with a step of 1 day. (B) The range of lag days with the highest correlations.

**Fig. 6:**
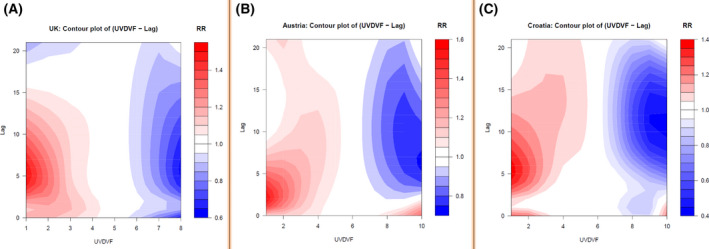
RR of COVID‐19 cases, relative to daily UVDVF, over 20 lag days for (A) the UK, (B) Austria, and (C) Croatia.

**Fig. 7:**
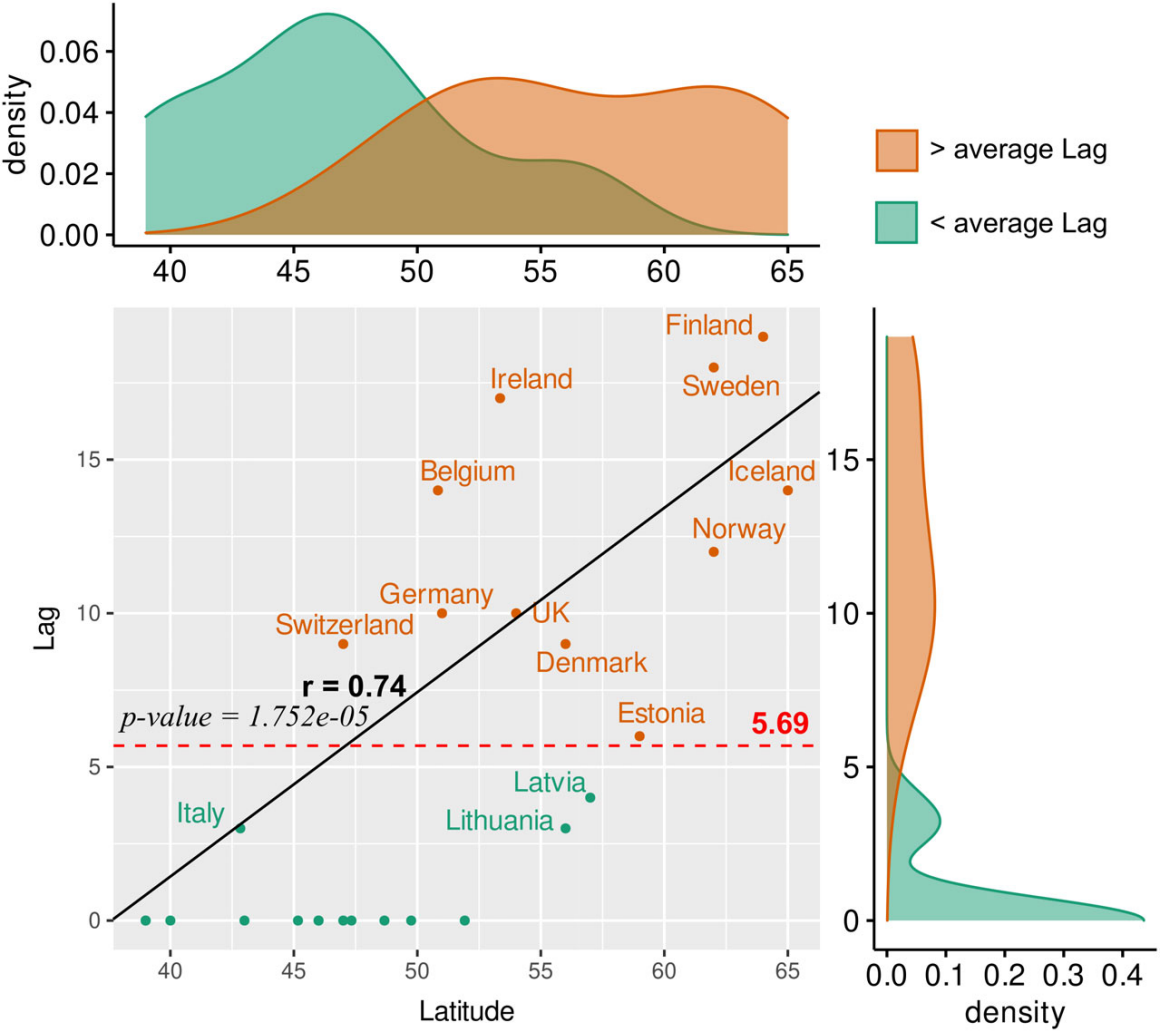
Association of lag days for UVDVF with latitude.

To understand the delayed effects of UV and the average temperature on the RR of COVID‐19, we performed exposure–lag–response association analysis using the DLNM approach. The results indicate that UVDVF had a significant delayed effect on COVID‐19 incidences, while the delayed impact of temperature could not be properly explained for all the European countries using this model as it shows insignificant distinct patterns for different countries. Figure S1 shows the RR pattern of COVID‐19 as a function of UVDVF and lag days by showing three‐dimensional plots of RR, UVDVF, and a lag of 21 days for 26 European countries. We observed that higher UVDVFs with different lag patterns are associated with a decreased RR of COVID‐19 cases in several countries. We observed an average lag of 3–10 days, with a higher UVDVF being associated with a reduction in RR. To further verify these results, we analyzed the distribution of the maximum Pearson correlation coefficients for temperature, UV index, and UVDVF versus COVID‐19 cases for all the countries, using different lags (Fig. 5A). We observed that the range of lags with the highest correlation was within 10 days, with an average value of 5.69 days (Fig. 5B). For a detailed explanation of the delayed effect of UVDVF, we used results from the UK, Austria, and Croatia as examples. Figure 6 depicts the lag effects of UVDVF on the RR of COVID‐19 incidence in (a) the UK, (b) Austria, and (c) Croatia. In this figure, the contour plots represent the change of RR with variation in UVDVF and lag days. From this plot, we can see that a higher UVDVF at different lag days is substantially associated with a reduction in RR. In the case of the UK, it can be seen that at the maximum UVDVF, the lowest RR was for the lag between 4 and 10 days (Fig. 6A). Similarly, in Austria, the minimum RR at 5–7 lag days was seen at the maximum UVDVF (Fig. 6B). In Croatia, at maximum UVDVF, 8–14 lag days were linked to a minimum RR (Fig. 6C). Furthermore, to understand the different lag patterns observed in the different countries, we performed a correlation analysis of lag days and the latitude of the countries. Interestingly, we observed a positive linear correlation of lag days with latitude (*r* = 0.74, *P*‐value = 1.752e^−05^; Fig. 7). This result is consistent with previous findings [54] and suggests that solar UV daily dosage, dependent on latitude, is an important parameter affecting plasma 25(OH) vitamin D levels in the populations of different countries, which have a significant impact on the RR of COVID‐19.

## Discussion

Recent evidence demonstrated that plasma 25(OH) vitamin D levels were significantly lowered in COVID‐19 patients, with an accompanying increase in COVID‐19 severity [55]. Vitamin D exerts an immunomodulatory role by increasing innate immune responses and secretion of antiviral peptides [56]. Higher plasma 25(OH) vitamin D levels were found to be associated with the likelihood of reduced survival and replication of viruses and also with reduced production of pro‐inflammatory cytokines, which impact the reduction in risk of COVID‐19 severity [57]. Hence, a lack of vitamin D in the body could cause immune dysfunction and increase the chances of COVID‐19 [58]. An association has been reported between vitamin D deficiency and higher COVID‐19 mortality rates in Europe [59]. Therefore, to understand the influence of vitamin D levels in the body on COVID‐19 risk in different countries, we performed a meta‐analysis based on available literature. We observed a negative association between low vitamin D levels in the body and an increase in COVID‐19 infection rates. Our meta‐analysis results suggest that a low vitamin D level is a crucial factor for the high risk of being afflicted with COVID‐19 across all populations.

Exposure to sunlight induces vitamin D production in the human body, which supports the hypothesis that lower COVID‐19 mortality rates in a population are associated with regular UV radiation exposure from sunlight [28]. The second wave of COVID‐19 infection in Europe that began at the beginning of autumn 2020 lends itself to the possibility that SARS‐CoV‐2 activity is associated with seasonal factors [3]. To test this hypothesis, we performed an analysis to understand the association of daily COVID‐19 cases with temperature, UV index, and UVDVF for 26 European countries. In all 26 countries, we observed that COVID‐19 cases are negatively associated with daily temperature, UV index, and UVDVF. However, the significant negative association of COVID‐19 cases with seasonal factors does not imply a cause‐and‐effect relationship. Daily temperature and UV doses should have delayed impacts on COVID‐19 cases. Therefore, to understand the lag effects of temperature and UV dose on the RR of COVID‐19, we performed an exposure–lag–response analysis using the DLNM approach. Our DLNM analysis indicated that UVDVF was significantly associated with the daily incidence of COVID‐19 with different patterns of time lags for different countries, with an average lag of 3–10 days, which supports the combined timescale of COVID‐19 incubation, testing, and reporting. A similar trend was observed in a study by Carleton *et al*. [60], where the authors used data from January 1, 2020, to April 10, 2020, from 173 countries and observed a lagged effect that peaked 9–11 days after UV exposure with heterogeneity in lag lengths being seen across the different countries. However, this variation in lag days differed from that reported in our study because of two main reasons. First, Carleton *et al*. [60] used only 3 months' worth of data, while we used 10 months' worth of data in our study. Secondly, these other authors used a temporal distributed lag regression model to infer the lag effect, while we used the DLNM approach in our study. However, the variation in the number of lag days in the two studies does not affect the conclusion that increasing UV exposure results in a significant reduction in COVID‐19 cases. To investigate the factors behind the different time lags for different countries, we found a strong correlation between latitude and lag days, which suggests that the variations in exposure–lag–response were due to the geographical locations of the countries. Furthermore, we observed that the COVID‐19 transmission rate decreased as UVDVF increased and that increasing UVDVF contributed to further decreases in the RR of COVID‐19. Therefore, our results suggest that UVDVF could guide spatial–temporal correlations of COVID‐19 cases in European countries and could be considered as a predictor for the incidence of COVID‐19.

Several recent studies also highlighted the impact of UV radiation on COVID‐19 incidence [60, 61, 62, 63, 64, 65]. Using machine‐learning approaches, Karapiperis *et al*. [62] demonstrated that UV radiation is an influential factor for COVID‐19 incidence rates, as compared to other factors, such as human mobility. Gunthe *et al*. [63] used 3 months' worth of data collected at the beginning of the COVID‐19 pandemic from 85 locations in different countries and observed that higher COVID‐19 levels were strongly dependent on the temperature and UV index. In a similar study, Asher *et al*. [64] observed higher COVID‐19 incidence in environments characterized by low temperature, dry air, and low UV radiation. However, the common limitation of these studies was a failure to consider the delayed effect of exposure to UV radiation on COVID‐19 reduction in different populations. Our results highlight that a low plasma 25(OH) vitamin D level, reflected as a mean of the average daily level of exposure to UV radiation, is a high‐risk factor for COVID‐19. Using a DLNM approach, we demonstrated an association of exposure–lag responses for different European countries. Furthermore, we found that lag responses to UV exposure was strongly correlated with the latitude of the European countries. A recent study observed an association of COVID‐19 cases with the latitude for 18 European countries and demonstrated its association with plasma 25(OH) concentration variation, suggesting that vitamin D is a contributing factor for COVID‐19 severity [54]. Therefore, the finding of this earlier report supports our results that variation in exposure–lag response is due to geographical location.

However, there are some limitations of the present study. The number of daily COVID‐19 cases reported in the different countries depends on the availability of testing and test accuracy. Furthermore, at the beginning of the COVID‐19 pandemic, the RT‐PCR test used required several days to return results. However, in the second part of 2020, a rapid antigen test was adopted, which can provide results on the same or the next day [66, 67, 68, 69]. Therefore, the record of positive cases on a particular day could be different due to the change in time associated with the use of a particular test, which could also affect the results of our study. Moreover, COVID‐19 transmission depends on several factors, such as public health interventions, population density, travel, lockdown, which were not accounted for in this study. We hypothesized that UV exposure is primarily associated with plasma 25(OH) vitamin D levels in the body. Our finding suggests that adequate daily UV exposure or vitamin D supplementation will be helpful in maintaining optimal vitamin D levels in the body, which could reduce the risk of contracting COVID‐19. However, food habits and dietary supplements could also be potential factors in determining individual vitamin D levels. Moreover, the relative contribution of seasonal UV exposure on plasma 25(OH) vitamin D levels is unknown.

## Conflict of interest

The authors declare no conflict of interest.

## Author contributions

SBM and MFM conceived and designed the project. SBM, AG, and EM acquired the data. SBM, AG, EM, EL, and SM analyzed and interpreted the data. SBM, AG, SM, and MFM wrote the paper. SBM, AG, SM, and MFM revised the article. All authors read and agreed on the final version of the article.

## Supporting information


**Fig. S1**. Three‐dimensional graphs showing the cumulative effects of UVDVF on daily confirmed COVID‐19 cases on different lag days.

**Table S1**. Daily COVID‐19 case numbers, 7‐day average of daily new cases, daily average temperature, UV index, and UVDVF for 26 European countries from March 1, 2020 to December 20, 2020. Downloaded raw data of temperature, UV index, and UVDVF from different stations are highlighted in the file. TAVG, UV_Avg, and UVDVF_Avg columns represent the respective daily average temperature, UV index, and UVDVF of all available stations for each country.

## Data Availability

The analyzed dataset generated during the study is included in the manuscript as Supporting Information.
