# Supplementary figures and images for: Seasonal UV exposure and vitamin D: association with the dynamics of COVID‐19 transmission in Europe
Source: FEBS Open Bio. 2021 Dec 4;12(1):106–17. doi: 10.1002/2211-5463.13309 (PMC8653358; doi:10.1002/2211-5463.13309)

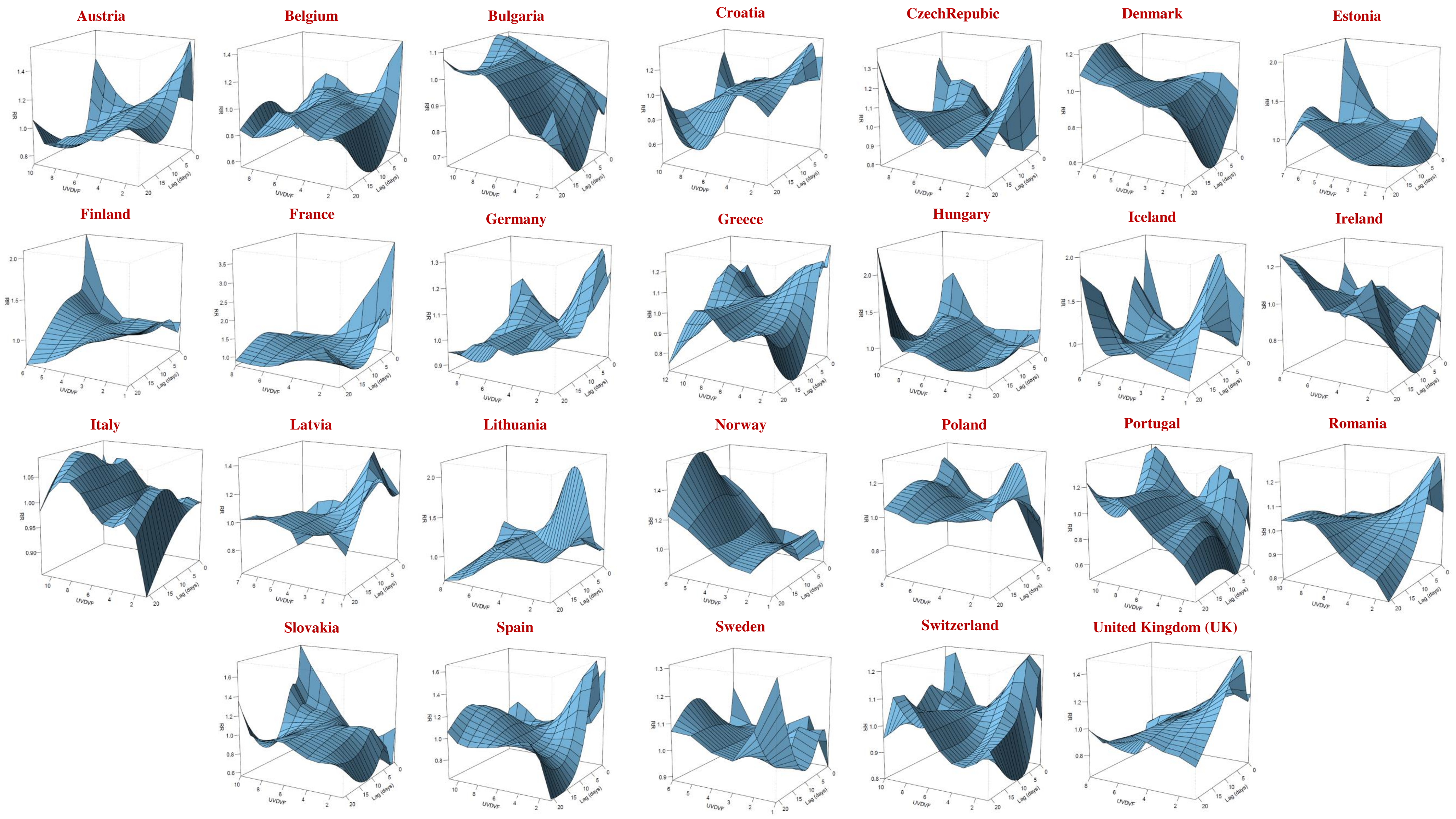

Supplement: Supplementary file 1 — Fig. S1. Three‐dimensional graphs showing the cumulative effects of UVDVF on daily confirmed COVID‐19 cases on different lag days. [file FEB4-12-106-s002.png]
